# The novel inflammatory biomarker GlycA and triglyceride-rich lipoproteins are associated with the presence of subclinical myocardial dysfunction in subjects with type 1 diabetes mellitus

**DOI:** 10.1186/s12933-022-01652-z

**Published:** 2022-11-24

**Authors:** Carlos Puig-Jové, Josep Julve, Esmeralda Castelblanco, M Teresa Julián, Núria Amigó, Henrik U Andersen, Tarunveer S Ahluwalia, Peter Rossing, Dídac Mauricio, Magnus T Jensen, Núria Alonso

**Affiliations:** 1 Department of Endocrinology & Nutrition, University Hospital Mútua de Terrassa, Terrassa, Spain; 2 Department of Medicine, Autonomous University of Barcelona (UAB), Barcelona, Spain; 3 Center for Biomedical Research on Diabetes and Associated Metabolic Diseases (CIBERDEM), Instituto de Salud Carlos III, Barcelona, Spain; 4 Sant Pau Biomedical Research Institute (IIB Sant Pau), Barcelona, Spain; 5 Division of Endocrinology, Metabolism and Lipid Research, Washington University School of Medicine in St. Louis, St Louis, MO USA; 6 Department of Endocrinology & Nutrition, University Hospital and Health Sciences Research Institute Germans Trias i Pujol, Badalona, Spain; 7 Biosfer Teslab SL, Reus, Spain; 8 Department of Basic Medical Sciences, Universitat Rovira i Virgili (URV), Reus, Spain; 9 Steno Diabetes Center Copenhagen, Herlev, Denmark; 10 Department of Biology, University of Copenhagen, Copenhagen, Denmark; 11 Department of Clinical Medicine, University of Copenhagen, Copenhagen, Denmark; 12 Faculty of Medicine, University of Vic - Central University of Catalonia (UVic/UCC), Vic, Spain; 13 Department of Endocrinology & Nutrition, Hospital de la Santa Creu i Sant Pau & Sant Pau Biomedical Research Institute (IIB Sant Pau), Barcelona, Spain; 14 Department of Cardiology, Copenhagen University Hospital Amager Hvidovre, Copenhagen, Denmark

**Keywords:** Myocardial dysfunction, Heart failure, Type 1 diabetes, Lipoproteins, GlycA

## Abstract

**Background:**

Subjects with Type 1 diabetes mellitus (T1DM) have an increased incidence of heart failure (HF). Several pathophysiological mechanisms have been involved in its development. The aim of this study was to analyze the potential contribution of the advanced lipoprotein profile and plasma glycosylation (GlycA) to the presence of subclinical myocardial dysfunction in subjects with T1DM.

**Methods:**

We included subjects from a Danish cohort of T1DM subjects (*Thousand & 1 study*) with either diastolic and/or systolic subclinical myocardial dysfunction, and a control group without myocardial dysfunction, matched by age, sex and HbA1c. All underwent a transthoracic echocardiogram and an advanced lipoprotein profile obtained by using the NMR-based Liposcale® test. GlycA NMR signal was also analyzed. Systolic dysfunction was defined as left ventricular ejection fraction ≤ 45% and diastolic dysfunction was considered as E/e′≥12 or E/e′ 8–12 + volume of the left atrium > 34 ml/m2. To identify a metabolic profile associated with the presence of subclinical myocardial dysfunction, a multivariate supervised model of classification based on least squares regression (PLS-DA regression) was performed.

**Results:**

One-hundred forty-six subjects had diastolic dysfunction and 18 systolic dysfunction. Compared to the control group, patients with myocardial dysfunction had longer duration of diabetes (p = 0.005), and higher BMI (p = 0.013), serum NTproBNP concentration (p = 0.001), systolic blood pressure (p < 0.001), albuminuria (p < 0.001), and incidence of advanced retinopathy (p < 0.001). The supervised classification model identified a specific pattern associated with myocardial dysfunction, with a capacity to discriminate patients with myocardial dysfunction from controls. PLS-DA showed that triglyceride-rich lipoproteins (TGRLs), such as VLDL (total VLDL particles, large VLDL subclass and VLDL-TG content) and IDL (IDL cholesterol content), as well as the plasma concentration of GlycA, were associated with the presence of subclinical myocardial dysfunction.

**Conclusion:**

Proatherogenic TGRLs and the proinflammatory biomarker Glyc A are strongly associated to myocardial dysfunction in T1DM. These findings suggest a pivotal role of TGRLs and systemic inflammation in the development of subclinical myocardial dysfunction in T1DM.

**Supplementary Information:**

The online version contains supplementary material available at 10.1186/s12933-022-01652-z.

## Background

Diabetes mellitus (DM) and heart failure (HF) are two multifaceted entities that involve high morbidity and mortality when both conditions coexist [1, 2]. The risk of HF is increased both in subjects with type 2 DM (T2DM) [3] and type 1 DM (T1DM) [4]. Indeed, DM is highly prevalent amongst patients with HF [5, 6], especially those with HF and preserved ejection fraction (HFpEF) [7, 8].

In population-based studies, the risk of HF in patients with diabetes (particularly T2DM) is significantly increased following adjustment for well-established HF risk factors [9]. The resulting specific form of cardiomyopathy is known as “diabetic cardiomyopathy” [10]. Although the concept of diabetic cardiomyopathy is often considered in individuals affected by T2DM, a metabolically-induced cardiomyopathy, independent of hypertension, nephropathy or ischemic heart disease is also evident in individuals with T1DM [11, 12].

Several pathophysiological mechanisms directly affecting the structure and function of the myocardium have been proposed to contribute to the development of diabetic cardiomyopathy. Among those described are hyperglycemia, hyperinsulinemia, inflammation and increased levels of circulating fatty acids (FAs) and triglycerides (TGs) [13–17]. Systemic inflammation plays a key role in HF etiopathogenesis [18, 19]. In that sense, GlycA has been described as a “composite biomarker of systemic inflammation” since its signal on nuclear magnetic resonance (NMR) spectra represents both the levels and degree of glycosylation of various acute phase proteins. GlycA NMR signal has been reported to be associated with increased risk of CV events, peripheral arterial disease, and mortality even after adjusting for other inflammatory markers [20, 21].

Lipotoxicity and cardiac lipid accumulation are other factors that have been related to the etiopathogenesis of diabetic cardiomyopathy [22]. In this line, myocardial metabolism studies have shown a reduced myocardial glucose uptake and an increased uptake of FAs in subjects with T1DM [23]. In T1DM, insulin deficiency promotes the mobilization of FAs from fat pads which results in an increased availability of excess FAs in different tissues, including the myocardium. When the capacity for storage and oxidation of mobilized FAs is exceeded, they can be transformed in other reactive species that further potentiates myocardial lipotoxicity. This cause of non-ischemic and non-hypertensive cardiomyopathy is often referred to as diabetic or “lipotoxic” cardiomyopathy.

Diabetic dyslipidaemia may also contribute to the diabetic myocardial dysfunction. Particularly, the excess flux of mobilized FAs to the liver promotes overproduction of TG-rich lipoproteins (TGRLs) and their remnants. Elevations in circulating TGRLs are frequently associated with increased concentrations of remnant cholesterol and with reduced high-density lipoproteins (HDL) cholesterol, and all contribute to the development of ischemic heart disease [24]. However, their contribution, if any, on non-ischemic cardiomyopathy remains poorly explored.

To the best of our knowledge, no previous studies in T1DM patients have studied the relationship between metabolic advanced profile with subclinical HF, defined as presence of impaired cardiac diastolic and/or systolic function without previous clinical manifestation of HF. Thus, the present study aims to analyze the contribution of inflammation (GlycA) and an advanced lipoprotein profile to the presence of subclinical myocardial dysfunction in a well-defined cohort of T1DM patients.

## Methods

### Study population

Our study population was selected from the *Thousand* & *1 study* cohort study. This study was carried out at the Steno Diabetes Center in Copenhagen (SDCC) with cardiology examinations conducted at the Department of Cardiology, Copenhagen University Hospital Herlev-Gentofte. It was conducted between April 1st, 2010, and April 1st, 2012, and was based on a large cohort of 1.093 patients with T1DM without known heart disease. Patients were included if they were 18 years of age or older, attending the outpatient clinic at the SDCC, diagnosed with T1DM, without known heart disease (defined as any known HF, coronary artery disease [including previous myocardial infarction, stable angina, previous percutaneous coronary intervention, or coronary artery bypass surgery], atrial fibrillation or atrial flutter, left bundle branch block, congenital heart disease, pacemaker or implantable cardioverter defibrillator implantation), and if willing to participate in the study. The study population has been described in detail elsewhere [25]. Briefly, from the total study population included in the study, 15.5% (n = 169) of the participants had grossly abnormal systolic or diastolic function.

From the original cohort of *Thousand* & *1 study*, we finally included a subgroup of 304 patients with T1DM, comprising 154 patients with myocardial dysfunction (the myocardial dysfunction group) and 150 controls. For the myocardial dysfunction group, we selected all T1DM patients who had either diastolic and/or systolic myocardial dysfunction and enough serum sample for conducting the NMR spectroscopy analysis. Additionally, we included 150 patients from the same cohort who had no echocardiographic alterations (the control group) matched by age, glycated hemoglobin (HbA1c) and gender.

### Ethical consideration

The original study was performed in accordance with the second Helsinki declaration and approved by the regional ethics committee (H-3-2009-139) and the Danish Data Protection Agency (00934-Geh-2010-003). All subjects gave written informed consent.

### Study visit

Prior to the echocardiographic examination, all patients received study information, signed the consent form and filled out a questionnaire with information about lifestyle factors, including smoking, exercise, alcohol consumption and cardiorespiratory symptoms. The use of cardiovascular treatments, such as lipid lowering medication (statins) and antihypertensive medication (beta blockers, calcium antagonists, diuretics, angiotensin-converting enzyme inhibitors or angiotensin II receptor antagonists), was also recorded. Blood pressure was measured in the supine position.

### Echocardiogram

Echocardiography was performed with a General Electric, Vivid 7 Dimension imaging system device (GE Vingmed Ultrasound AS, Horten, Norway) with a 3.5-MHz transducer in accordance with the recommendations from the European Association of Echocardiography/American Society of Echocardiography [26]. Echocardiographic examinations were read and analysed using the General Electric EchoPAC software (BT11), recording three consecutive heart cycles. Left ventricle ejection fraction (LVEF) was determined by Simpson’s biplane method. Left atrial volume (LAV) was determined by the recommended biplane area-length method and indexed for body surface area. LV mass was determined by the linear method and indexed for body surface area.

Subclinical myocardial dysfunction was defined when patients had systolic and/or diastolic myocardial dysfunction on the echocardiogram in the absence of HF symptoms. Systolic dysfunction was defined as LVEF ≤ 45% determined by Simpson´s biplane method and diastolic dysfunction was considered if there was evidence of long-standing LV filling pressure defined as E/e′ >12 (where E is diastolic mitral early inflow velocity and e′ is pulsed-wave early diastolic tissue doppler velocity) or E/e′ 8–12 and LAV > 34 ml/m2.

### Biochemistry

Information about biochemistry such as HbA1c, p-creatinine and albuminuric status was collected from electronic patient files at the SDCC from the ambulatory visit closest to study inclusion, which was maximally ± 4 months from inclusion. This information was collected after analyzing the echocardiography.

The urinary albumin excretion rate (UAER) was measured in 24-h sterile urine collections by enzyme immunoassay. Patients were categorized as normoalbuminuric if the UAER, in two out of three consecutive measurements, was < 30 mg/24 h, microalbuminuric if the UAER was between 30 and 300 mg/24 h, and macroalbuminuric if the UAER > 300 mg/24 h. HbA1c was measured by high-performance liquid chromatography (normal range: 21–46 mmol/mol [4.1–6.4%]; Variant; Bio-Rad Laboratories, Munich, Germany), and serum creatinine concentration was measured by an enzymatic method (Hitachi 912; Roche Diagnostics, Mannheim, Germany). The estimated glomerular filtration rate (eGFR) was calculated by the MDRD method.

### Lipoprotein and glycoprotein analysis by NMR spectroscopy (advanced profile)

Serum samples were shipped on dry ice from the SDCC to the Biosfer Teslab facilities (Reus, Spain) for Liposcale® lipoprotein and glycoprotein analysis. Samples were kept at −80°C until the NMR analysis. 200 µl of serum was diluted with 50 µl deuterated water and 300 µl of 50 mM phosphate buffer solution at pH 7.4. 1H-NMR spectra were recorded at 306 K on a Bruker Avance III 600 spectrometer operating at a proton frequency of 600.20 MHz (14.1 T).

### Lipoprotein analysis

Lipoprotein profiling was obtained by using the Liposcale® test (IVD-CE), a previously reported method based on a two-dimensional ^1^H-NMR diffusion-ordered spectroscopy (DOSY) approach for lipoprotein profile characterization including lipid content (cholesterol and triglyceride concentration), size and particle number of the main lipoprotein classes [27]. The methyl signal was deconvoluted by using 9 lorentzian functions to determine the lipid concentration of the large, medium and small subclasses of the main lipoprotein classes: very low-density lipoproteins (VLDL), low-density lipoproteins (LDL) and HDL, and their size associated diffusion coefficients. Then, the lipid concentration was combined with their associated particle volume in order to quantify the number of particles required to transport the measured lipid concentration of each lipoprotein subclass. Weighted average VLDL, LDL and HDL particle sizes were calculated from various subclass concentrations by summing the known diameter of each subclass multiplied by its relative percentage of subclass particle number. The variation coefficients for the particle numbers were between 2% and 4%, and for the particle sizes they were lower than 0.3%.

### Glycoprotein analysis

The region of the 1H-NMR spectrum where the glycoproteins resonate (2.15–1.90 ppm) was deconvoluted using several analytical functions associated with specific plasmatic sugar-protein bonds according to a previously published procedure [28]. For each function, the total area (proportional to concentration), height, position and bandwidth were determined. The area of the specific glycoprotein signal defined as the GlycA NMR signal arose from the acetyl groups of N-acetylglucosamine and N-acetylgalactosamine bonded to plasmatic proteins [29]. Consistent with that, a larger GlycA area reflects a higher level of plasmatic glycosylation. The height-to-width (H/W) ratio of the GlycA signal, associated with its molecular aggregation state, was also reported. The H/W parameter, increased during inflammatory processes, reflects the sugar-protein bond flexibility, indicating glycosylation in accessible regions of the proteins. Height is defined as the difference from baseline to the maximum of the corresponding NMR peaks and the width value corresponds to the peak width at half height.

### Statistical analysis

We used widely described chemometric methods [30] in order to identify a specific lipoprotein/glycoprotein profile associated with myocardial dysfunction. Briefly, multivariate statistical analyses were computed in MATLAB, Ver. 7.10.0 using PLS-Toolbox, Ver. 5.2.2 (Eigenvector Research Inc., Manson, WA, United States) after application of a genetic algorithm (GA) for variable selection to optimize the predictive ability of the model. Partial least squares discriminant analysis (PLS-DA) models were used as a supervised classification method between the study groups. This is a well established and widely used method in chemometrics- and metabolomics-based analyses when a large number of variables are used to classify two-stage conditions. PLS-DA relates the X matrix (input data including NMR derived parameters, clinical and anthropometric variables), and the Y matrix (presence or absence of myocardial disfunction) to find the maximum discrimination between classes (study groups). The PLS-DA method reduces the dimensionality of the initial dataset (X matrix), creating a new multidimensional dataset for each individual maximizing the total variance of data using just a few components (the latent scores), obtained from the specific contribution of each variable (loadings) to the new multidimensional axis [31].

On the other hand, to avoid overfitting and correct the multiple testing effects, we auto scaled and cross-validated by the permutation Venetian Blinds cross validation method by using 10 splits of the input data. The area under the curve (AUC) was used to evaluate the capacity of the NMR and clinical variables to distinguish between the two groups (with and without subclinical myocardial dysfunction).

The following subset of 19 clinical and biochemical variables was selected after a GA approach: diabetes duration, body mass index (BMI), hemoglobin, intermediate-density lipoproteins (IDL) cholesterol content (IDL-C), LDL cholesterol content (LDL-C), VLDL TG content (VLDL-TG), total VLDL particles (VLDL Particles), Large VLDL, total LDL particles (LDL Particles), total HDL particles (HDL Particles); Large HDL, Medium HDL, LDL size (LDL-Z), HDL size (HDL-Z), GlycA area, H/W GlycA ratio (H/W GlycA), HDL-TG/HDL-C ratio (HDL ratio), Small VLDL/total VLDL particles ratio (% Small VLDL) and Small LDL/total LDL particles ratio (% Small LDL).

Finally, we assessed the discrimination capacity of NMR biomarkers to predict the presence of myocardial dysfunction when these variables were added to the model including only traditional clinical variables (age, sex, eGFR, NTproBNP, BMI, diabetes duration and systolic blood pressure) by using logistic regression.

## Results

### Clinical characteristics of our study population

Our study population comprised a total of 304 T1DM subjects (53.6% women, 50.7% subjects with myocardial dysfunction) with a median [min;max] age of 62.1 [22.5;87.0] years at inclusion, median diabetes duration of 33 years and a median HbA1c value of 8.0%. Clinical characteristics by group are shown in Table 1. Briefly, there were no differences regarding gender, age, HbA1c value, smoking habit, total cholesterol, LDL cholesterol and TGs values between the groups. From a total of 154 subjects with myocardial dysfunction, 146 (94.8%) and 18 (11.7%) had diastolic and systolic dysfunction, respectively. Subjects with myocardial dysfunction showed, in comparison with controls, a longer diabetes duration (35.1 ± 14.9 years vs. 30.1 ± 15.5 years; p = 0.005), a higher BMI (26.1 ± 3.9 kg/m^2^ vs. 25.0 ± 3.7 kg/m^2^; p = 0.013), a higher systolic blood pressure (143 mmHg vs. 136 mmHg; p < 0.001) and a lower eGFR (75.4 ± 26.2 mL/min/1.73m^2^ vs. 83.7 ± 21.0 mL/min/1.73m^2^; p = 0.003). Differences in echocardiographic parameters and serum cardiac biomarkers of HF were also observed between the two groups. In summary, subjects with myocardial dysfunction had lower mean (SD) ejection fraction (55.8 ± 7.5% vs. 58.6 ± 5.2%, p < 0.001), higher LAV (34.1 ± 7.6 ml/m^2^ vs. 28.9 ± 5.5 ml/m^2^, p < 0.001) and higher median [25th;75th] concentrations of N-terminal fragment of pro-B-type natriuretic peptide (NTproBNP) (352 [163;591] pg/mL vs. 249 [116;449] pg/mL, p = 0.001), and mid-regional pro atrial natriuretic peptide (MRproANP) (100 [69.2;144] pmol/L vs. 82.3 [56.4;112] pmol/L, p < 0.001). Also, the percentage of subjects with E/e′ >8 on echocardiography was higher in subjects with myocardial dysfunction compared with those without myocardial dysfunction (94.8% vs. 38.7%, p < 0.001). Additionally, the use of statin and antihypertensive therapies was more frequent in subjects with myocardial dysfunction than in those without (p = 0.039 and p < 0.001 respectively). Finally, subjects with myocardial dysfunction were more likely to have advanced stages of retinopathy and albuminuria (p < 0.001 for both comparisons) than those without.

**Table 1:** Descriptive analysis of clinical variables by group

Variable	Myocardial Dysfunction (n 154)	Control (n 150)	p value
**Sex (men)**	72 (46.8%)	69 (46.0%)	0.987
**Age (years)**	61.0 (11.7)	60.6 (11.1)	0.756
**Diabetes duration (years)**	35.1 (14.9)	30.1 (15.5)	0.005
**EF (%)**	55.8 (7.58)	58.6 (5.24)	< 0.001
**E/e´ Cocient**			< 0.001
**E/e´ <8**	8 (5.19%)	92 (61.3%)	
**E/e´ 8–12**	67 (43.5%)	58 (38.7%)	
**E/e´ >12**	79 (51.3%)	0 (0.00%)	
**LAV (ml/m2)**	34.1 (7.67)	28.9 (5.52)	< 0.001
**Diastolic HF**			< 0.001
**No**	8 (5.19%)	150 (100%)	
**Yes**	146 (94.8%)	0 (0.00%)	
**Systolic HF**			< 0.001
**No**	136 (88.3%)	150 (100%)	
**Yes**	18 (11.7%)	0 (0.00%)	
**Height (m)**	1.71 (0.09)	1.73 (0.10)	0.037
**Weight (Kg)**	76.4 (15.1)	75.1 (13.7)	0.422
**BMI (Kg/m2)**	26.1 (3.93)	25.0 (3.66)	0.013
**Diastolic BP (mmHg)**	73.5 (11.2)	71.4 (9.53)	0.073
**Systolic BP (mmHg)**	143 (17.7)	136 (17.9)	0.002
**Statin use**			0.039
**No**	52 (33.8%)	69 (46.0%)	
**Yes**	102 (66.2%)	81 (54.0%)	
**Antihypertensive use**			< 0.001
**No**	29 (19.8%)	59 (39.3%)	
**Yes**	125 (81.2%)	91 (60.7%)	
**Smoking habit**			0.140
**Never smoker**	51 (33.1%)	61 (40.7%)	
**Current smoker**	25 (16.2%)	30 (20.0%)	
**Ex-smoker**	78 (50.6%)	59 (39.3%)	
**NTproBNP (pg/mL)**	352 [163;591]	249 [116;449]	< 0.001
**MRproANP (pmol/L)**	100 [69.2;144]	82.3 [56.4;112]	< 0.001
**HbA1c value (%)**	8.19 (1.11)	8.01 (1.20)	0.178
**Total cholesterol (mmol/L)**	4.77 (0.99)	4.72 (0.93)	0.620
**LDL cholesterol (mmol/L)**	2.44 (0.70)	2.44 (0.80)	0.997
**Triglycerides (mmol/L)**	1.12 (0.78)	1.07 (0.60)	0.595
**24 h albumin in urine (mg/24 h)**	309 (1587)	18.0 (26.0)	0.026
**eGFR value (mL/min/1.73m2)**	75.4 (26.2)	83.7 (21.0)	0.003
**Albuminuria status**			< 0.001
**Normoalbuminuria**	73 (47.4%)	110 (73.3%)	
**Microalbuminuria**	42 (27.3%)	35 (23.3%)	
**Macroalbuminuria**	39 (25.3%)	5 (3.33%)	
**Retinopathy status global (worst eye)**			< 0.001
**Normal**	37 (24.2%)	55 (36.7%)	
**Simplex retinopathy**	63 (41.2%)	79 (52.7%)	
**Proliferative retinopathy**	53 (34.6%)	16 (10.7%)	

Results are expressed in mean (SD) for continuous variables, frequency and percentage for categorical variables and median [25th;75th] for variables not normally distributed. EF: ejection fraction, E/e´: estimated left ventricular filling pressure, LAV: left atrial volume, HF: heart failure. BMI: body mass index, BP: blood pressure, NTproBNP: N-terminal fragment of pro-B-type natriuretic peptide, MRproANP: Mid-regional pro atrial natriuretic peptide, HbA1c: glycated hemoglobin, LDL: low-density lipoproteins, eGFR: estimated glomerular filtration rate

### Lipoprotein and glycoprotein NMR advanced profile in subjects with myocardial dysfunction and controls

Results from the lipoprotein and glycoprotein profile are shown in Table 2. Most remarkably, compared with controls, subjects with myocardial dysfunction presented a more proatherogenic and pro-inflammatory profile, associated with increased IDL cholesterol and TG content (p = 0.004 and 0.003 respectively) and a greater GlycA area (p < 0.001). The LDL lipid composition, defined as LDL-TG / LDL-C, was also increased in the myocardial dysfunction group, reflecting increased remnant levels and neutral lipid heteroexchange (TGs and cholesteryl esters) from TG-rich particles to LDL particles, and vice versa, by the cholesteryl ester transfer protein.

**Table 2:** Lipoprotein and glycoprotein NMR advanced profile in cases and controls

Variable	Myocardial Dysfunction (n 154)	Control (n 150)	p value
**VLDL-P (nmol/L)**	41.3 (26.5)	44.4 (30.3)	0.343
**Large VLDL-P**	1.11 (0.65)	1.17 (0.65)	0.406
**Medium VLDL-P**	3.66 (2.31)	4.08 (3.53)	0.215
**Small VLDL-P**	36.5 (24.5)	39.1 (27.0)	0.377
**LDL-P (nmol/L)**	1217 (231)	1230 (235)	0.608
**Large LDL-P**	172 (32.7)	173 (30.7)	0.757
**Medium LDL-P**	353 (115)	363 (109)	0.457
**Small LDL-P**	691 (134)	694 (138)	0.845
**HDL-P (nmol/L)**	32.6 (6.59)	32.4 (6.38)	0.708
**Large HDL-P**	0.29 (0.05)	0.29 (0.04)	0.891
**Medium HDL-P**	11.2 (2.55)	11.0 (2.26)	0.451
**Small HDL-P**	21.1 (4.58)	21.1 (4.61)	0.895
**Total-P/HDL-P**	40.1 (11.5)	41.3 (14.2)	0.442
**LDL-P/HDL-P**	38.8 (10.9)	39.8 (13.3)	0.465
**VLDL-C (mg/dL)**	15.2 (10.80)	13.8 (9.35)	0.198
**IDL-C (mg/dL)**	11.6 (4.98)	10.1 (3.96)	0.004
**LDL-C (mg/dL)**	120 (22.80)	119 (23.40)	0.813
**HDL-C (mg/dL)**	65.1 (16.40)	66.7 (17.80)	0.401
**VLDL-TG (mg/dL)**	57.8 (39.30)	53.9 (33.10)	0.353
**IDL-TG (mg/dL)**	11.9 (3.90)	10.7 (3.25)	0.003
**LDL-TG (mg/dL)**	16.5 (4.46)	15.5 (4.15)	0.048
**HDL-TG (mg/dL)**	18.2 (4.83)	17.2 (4.98)	0.073
**VLDL-Z (nm, diameter)**	41.9 (0.44)	42.0 (0.45)	0.921
**LDL-Z (nm, diameter)**	21.0 (0.31)	21.0 (0.34)	0.687
**HDL-Z (nm, diameter)**	8.28 (0.07)	8.28 (0.08)	0.633
**Cholesterol total (mg/dL)**	212 (31.2)	210 (30.60)	0.582
**TG total (mg/dL)**	104 (47.1)	97.2 (39.40)	0.155
**HDL-C (mg/dL)**	65.1 (16.4)	66.7 (17.80)	0.401
**Ratio VLDL (VLDL-TG/ VLDL-C)**	4.19 (1.34)	4.41 (1.41)	0.160
**Ratio LDL (LDL-TG/ LDL-C)**	0.14 (0.03)	0.13 (0.03)	0.012
**Ratio IDL (IDL-TG/IDL-C)**	1.08 (0.18)	1.12 (0.23)	0.123
**Ratio HDL (HDL-TG/ HDL-C)**	0.29 (0.10)	0.27 (0.10)	0.061
**% VLDL (Small/total VLDL-P)**	0.88 (0.04)	0.88 (0.04)	0.916
**% LDL (Small/total LDL-P)**	0.57 (0.06)	0.57 (0.06)	0.491
**% HDL (Small/total HDL-P)**	0.65 (0.04)	0.65 (0.05)	0.596
GlycA area (1,39*10 ^**2**^ **µmol/L)**	4.93 (0.94)	4.66 (0.85)	0.009
** H/W GlycA**	16.9 (3.09)	15.8 (2.80)	0.001

Results are expressed in mean (SD). VLDL: very-low-density lipoproteins, P: particles, LDL: low-density lipoproteins, HDL: high-density lipoproteins, C: cholesterol, IDL: intermediate-density lipoproteins, TG: triglycerides, Z: size, H/W: height-to-width ratio

### Lipoprotein and glycoprotein signature associated with the presence of myocardial dysfunction

**Fig. 1:**
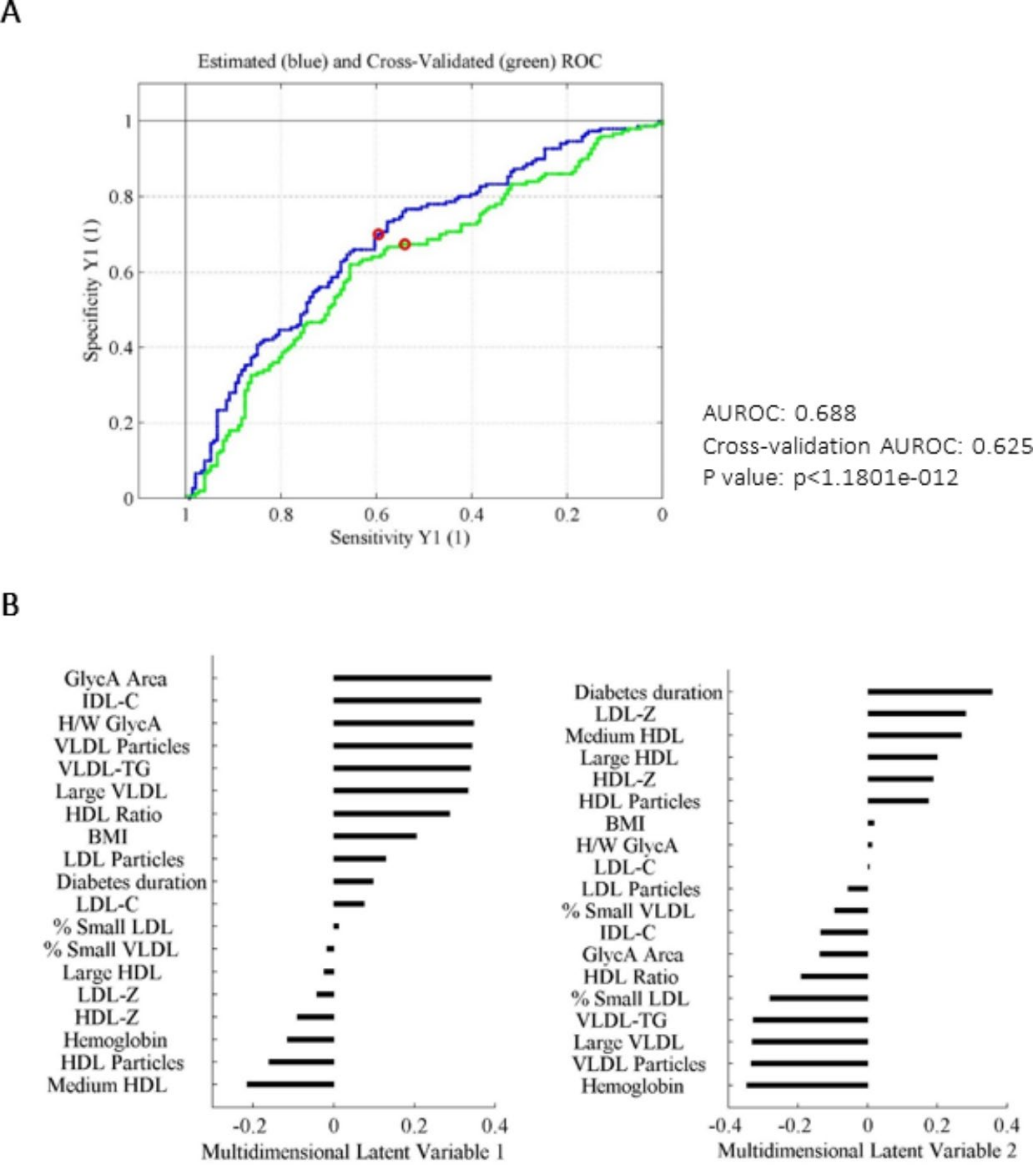
** A**: Estimated and cross-validated ROC curve of the PLS-DA classification model. **B**: Contribution of each variable to the multidimensional latent variables LV1 and LV2. PLS-DA relates the X matrix (input data including NMR derived parameters, clinical and anthropometric variables) and the Y matrix (presence or absence of myocardial disfunction) to find the maximum discrimination between both groups. AUROC: area under the ROC curve, PLS-DA: partial least squares discriminant analysis, LV: latent variable, NMR: nuclear magnetic resonance

We used a multivariant classification approach based on PLS-DA in order to identify a specific lipoprotein and glycoprotein profile associated with myocardial dysfunction in subjects with T1DM. Figure 1 shows the good performance of the classification method by using a ROC curve analysis, and the contribution of each variable to the model, summarized in the loadings plot of the principal two latent multidimensional variables (LV1 and LV2).

This supervised classification model succeeded in identifying a specific pattern associated with myocardial dysfunction, with a capacity to discriminate diabetic patients with myocardial dysfunction from the rest of the individuals without CVD, in a modest but significant way in relation to fortuitously obtaining the correct classification (area under the ROC curve 0.63, p < 1.1801e-012).

In that sense, we used the PLS-DA classification approach to investigate which variables discriminated best between subjects with myocardial dysfunction and controls (non-myocardial dysfunction subjects). LV1 showed that the variables with the most significant contribution to explain the presence of myocardial dysfunction in T1DM subjects beyond well-established risk factors included: NMR-determined GlycA area and H/W GlycA ratio as well as TGRLs, such as VLDL (total VLDL particles, large VLDL subclass and VLDL-TG content) and IDL (IDL cholesterol content). In the second latent variable (i.e. after the first discriminating analysis based on inflammatory parameters and VLDL and IDL related parameters was accomplished), other clinical variables including diabetes duration and LDL related characteristics (LDL-Z) became discriminative, whereas VLDL related variables were no longer discriminant. Furthermore, we compared the predicted probability of the presence of myocardial dysfunction when NMR variables were added together with traditional risk factors by using logistic regressions. Supplementary Fig. 1 shows the predicted probability of myocardial dysfunction in a model (model 1) including only classical risk variables (age, sex, eGFR, NTproBNP, BMI, diabetes duration and systolic blood pressure > 140mmHg). We built a second model (model 2) including both classical variables and the NMR-assessed biomarkers. The inclusion of the NMR variables in this second model significantly increased the area under the ROC curve from 0.62 [0.56–0.68] to 0.67 [0.61–0.73], with a net reclassification analysis improvement considering NMR-assessed parameters of 21%.

## Discussion

To our knowledge, this is the first study to date evaluating the association between a metabolic advanced profile with subclinical myocardial dysfunction assessed with echocardiography in subjects with T1DM. The analysis has been performed in a well characterized large cohort of T1DM patients without prior CVD. Our data showed that the inflammation biomarker GlycA as well as VLDL-related variables (total VLDL particles, large VLDL subclass and VLDL-TG content) and IDL-related variables (IDL cholesterol content) were associated with the presence of myocardial dysfunction in T1DM subjects.

Although the underlying mechanisms explaining the myocardial dysfunction remain still obscure, accumulating evidence supports the role of chronic inflammation in its manifestation and progress to HF [32–33]. In support of this, our data suggest that the circulating levels of GlycA are related to myocardial dysfunction in T1DM subjects. Our finding is consistent with the significance of circulating GlycA, as it integrates in a single metrics the plasma concentrations [34] and glycosylation states [29, 35] of several abundant acute-phase inflammatory proteins. Notably, GlycA exhibits lower intraindividual variability and greater analytical precision than other inflammatory markers [34], thereby suggesting a role as a potential novel biomarker linked to systemic inflammation and CVD assessment [36–38]. In line with this, the serum levels of GlycA are directly associated with those of C-reactive protein (CRP) [39], which is a well-established diagnostic biomarker of inflammation used in clinical practical clinical. Noteworthy, the advantage offered by GlycA over CRP is that it may integrate multiple inflammatory pathways by capturing the global signal of several proteins and, therefore, better captures the degree of systemic inflammation [40]. In addition, the measurement of GlycA is associated with higher reliability and lower intra-individual variability than CRP because it is detected similarly in both serum and plasma samples, in fasting and non-fasting states, after short or long-term storage [34], and also after repeated intraindividual determinations over time [41].

A main finding of our study was that GlycA values allowed identification of T1DM subjects with and without subclinical myocardial dysfunction. Our data are in line with other study that support the concept that GlycA is associated with an increased risk of HF, particularly HFpEF, which is independent of traditional CVD risk factors and other inflammatory markers [42]. To our knowledge, data about GlycA concentrations in subjects with T1DM are lacking and for the first time our results regarding GlycA further suggest a role for inflammatory mechanisms in the pathogenesis of myocardial dysfunction in these patients.

Our data also showed that some characteristics of VLDL-related particles, together with GlycA, could help to better discriminate between T1DM subjects with and without subclinical myocardial dysfunction. Regarding T1DM, the advanced characteristics of VLDL has been positively and independently associated with arterial stiffness [43]. Likewise, similar associations have also been described in T1DM women with previous pre-eclampsia with carotid atherosclerosis [44].

Additionally, the role of TGRLs in HF has been previously established. In two prospective studies of 113,554 individuals from the general population in Denmark, a higher risk of HF for stepwise higher non-fasting TGs (i.e., TGRLs, which also include the chylomicron remnants) was found [45].

Although evidence supporting a relationship between cholesterol remnants and non-ischemic causes of HF is limited, the induction of VLDL receptor (VLDLR) abundance and TGRL uptake by cardiomyocytes in an experimental postprandial setting [46] suggest a role for VLDLR in atrial cardiomyopathy and ventricular dysfunction [47, 48]. Indeed, an association of atrial fibrillation with abnormal VLDL-related lipid metabolism has been suggested by some authors [48] and an increased risk of developing atrial fibrillation among patients with T1DM compared with the general population has been reported [49].

According to previous studies, the circulating baseline levels of GlycA exhibit significant positive associations with incident CVD event rates and associated mortality [37]. Remarkably, such correlations remain significant even after adjusting for several other established CVD risk factors. In our study, the discriminating capability of our PLS-DA model was not improved when including either the 24 h-urinary albumin excretion rate or NTproBNP as input variables, maintaining GlycA as the best discriminating variable for the presence of myocardial disfunction in subjects with T1DM (data not shown).

The multivariant approach used in the present study by using the PLS-DA methodology helped in identifying concomitant lipoprotein characteristics (TGRLs) that, together with the proinflammatory GlycA, could mainly account for myocardial dysfunction in T1DM subjects on top of the commonly used traditional and well-established risk factors, including BMI, gender, HbA1c or diabetes duration. Inflammation showed the higher discriminant ability distinguishing myocardial dysfunction among T1DM patients, with the GlycA area being the highest contributor to the first multidimensional latent variable (LV1). Of note, diabetes duration helped to discriminate myocardial dysfunction from non-myocardial dysfunction T1DM patients only once the inflammatory signature was considered, as shown in the second latent variable (LV2) of the multivariable classification analysis. Furthermore, this second multidimensional latent variable showed a loss of association between isolated increased TG-rich parameters and myocardial dysfunction. Therefore, our data revealed an interaction between GlycA and TGRLs in T1DM subjects with myocardial dysfunction. This association was lost in those patients without myocardial dysfunction.

Our study had some limitations that should be taken into account. We matched individuals with and without subclinical myocardial dysfunction by age, sex and glycemic control. This may have resulted in a biased comparison because, according to original *Thousand* & *1 study* results, patients with myocardial dysfunction were likely to be older and have longer diabetes duration than subjects with normal function. Therefore, by selecting subjects who were similar for these criteria, at least one group may be unrepresentative of the population from which it came, and the abnormalities observed in the NMR profile may be different from a truly representative population. Furthermore, considering the condition of hypertension as a risk factor for myocardial dysfunction, is important to emphasize that the use of antihypertensive therapies was significantly more frequent in the group of subjects with myocardial dysfunction. Nevertheless, due to the fact that these drugs can be used both as an antihypertensive treatment as well as a nephroprotective intervention, we consider this as a limitation to assess the condition of hypertension associated with myocardial dysfunction. Additionally, the small number of subjects who presented systolic dysfunction within subjects with myocardial dysfunction (n = 18, 11.7%) implies that the results obtained in the present study should be primordially referred to diastolic dysfunction. Finally, since this study is cross-sectional we cannot infer causality between the metabolic profile and presence of myocardial dysfunction. Possible strengths should also be mentioned, since the current population is selected from a large study of ambulatory T1DM patients without previous known heart disease. This cohort had the advantage of a relatively low HbA1c and may therefore be at a lower risk of HF than typical T1DM patients and that may potentially have strengthened our findings.

## Conclusion

The present study uncovered associations between subclinical myocardial dysfunction and advanced NMR metabolic characteristics in patients with T1DM that were hidden in conventional analyses. The GlycA area and the H/W GlycA ratio, as well as TGRLs (VLDL and IDL) related variables, were revealed as strong contributors of subclinical myocardial dysfunction. According to the aforementioned results, we propose a pivotal role of TGRLs characteristics and systemic inflammation reflected by the GlycA biomarker in subclinical myocardial dysfunction in T1DM patients.

Estimation of the presence of subclinical myocardial dysfunction among T1DM patients and its relationship with lipoprotein and glycoprotein characteristics revealed in this study still deserves more attention in the future. Therefore, further studies will be required to clarify the potential clinical applications of these findings as well as to investigate their biological basis.

## Electronic supplementary material

Below is the link to the electronic supplementary material.

Supplementary Material 1: Supplementary Fig. 1: Comparison of the distribution of the predicted probabilities showing the classification performance of the presence of MCD between two different models, being the x-axis the predicted probabilities for both classes and the y-axis the count of observations. A: Model 1 includes classical risk variables (age, sex, eGFR, NTproBNP, BMI, diabetes duration and systolic blood pressure >140mmHg). B: Model 2 includes classical risk variables and the NMR-assessed biomarkers. The inclusion of NMR parameters significantly increased the AUROC from 0.62 [0.56–0.68] to 0.67 [0.61–0.73], with a NRI considering NMR-assessed parameters of 21%. MCD: myocardial dysfunction, BMI: body mass index, NMR: nuclear magnetic resonance, AUROC: area under the ROC curve, NRI: net reclassification improvement.

## Data Availability

Primary material is held by the authors.

## Abbreviations

DM: Diabetes mellitus.
HF: Heart failure.
T2DM: Type 2 diabetes mellitus.
T1DM: Type 1 diabetes mellitus.
HFpEF: Heart failure with preserved ejection fraction.
CVD: Cardiovascular disease.
FAs: Fatty acids.
TGs: Triglycerides.
NMR: Nuclear magnetic resonance.
TGRLs: Triglycerides-rich lipoproteins.
HDL: High density lipoproteins.
SDCC: Steno diabetes center in Copenhagen.
HbA1c: Glycated hemoglobin.
LVEF: Left ventricle ejection fraction.
LAV: Left atrial volume.
UAER: Urinary albumin excretion rate.
eGFR: Estimated glomerular filtration rate.
DOSY: Diffusion-ordered spectroscopy.
VLDL: Very low-density lipoproteins.
LDL: Low-density lipoproteins.
GA: Genetic algorithm.
PLS-DA: Partial least square discriminant analysis.
AUC: Area under the curve.
BMI: Body mass index.
IDL: Intermediate-density lipoprotein.
NTproBNP: N-terminal fragment of pro-B-type natriuretic peptide.
MRproANP: Mid-regional pro atrial natriuretic peptide.
CRP: C-reactive protein.
VLDLR: VLDL receptor.
hsCRP: High-sensitivity CRP.
