# Supplementary material for: The novel inflammatory biomarker GlycA and triglyceride-rich lipoproteins are associated with the presence of subclinical myocardial dysfunction in subjects with type 1 diabetes mellitus
Source: Cardiovasc Diabetol. 2022 Nov 24;21:257. doi: 10.1186/s12933-022-01652-z (PMC9700974; doi:10.1186/s12933-022-01652-z)
Supplement: Supplementary file 1 — Supplementary Material 1: Supplementary Fig. 1: Comparison of the distribution of the predicted probabilities showing the classification performance of the presence of MCD between two different models, being the x-axis the predicted probabilities for both classes and the y-axis the count of observations. A: Model 1 includes classical risk variables (age, sex, eGFR, NTproBNP, BMI, diabetes duration and systolic blood pressure >140mmHg). B: Model 2 includes classical risk variables and the NMR-assessed biomarkers. The inclusion of NMR parameters significantly increased the AUROC from 0.62 [0.56–0.68] to 0.67 [0.61–0.73], with a NRI considering NMR-assessed parameters of 21%. MCD: myocardial dysfunction, BMI: body mass index, NMR: nuclear magnetic resonance, AUROC: area under the ROC curve, NRI: net reclassification improvement. [file 12933_2022_1652_MOESM1_ESM.docx]

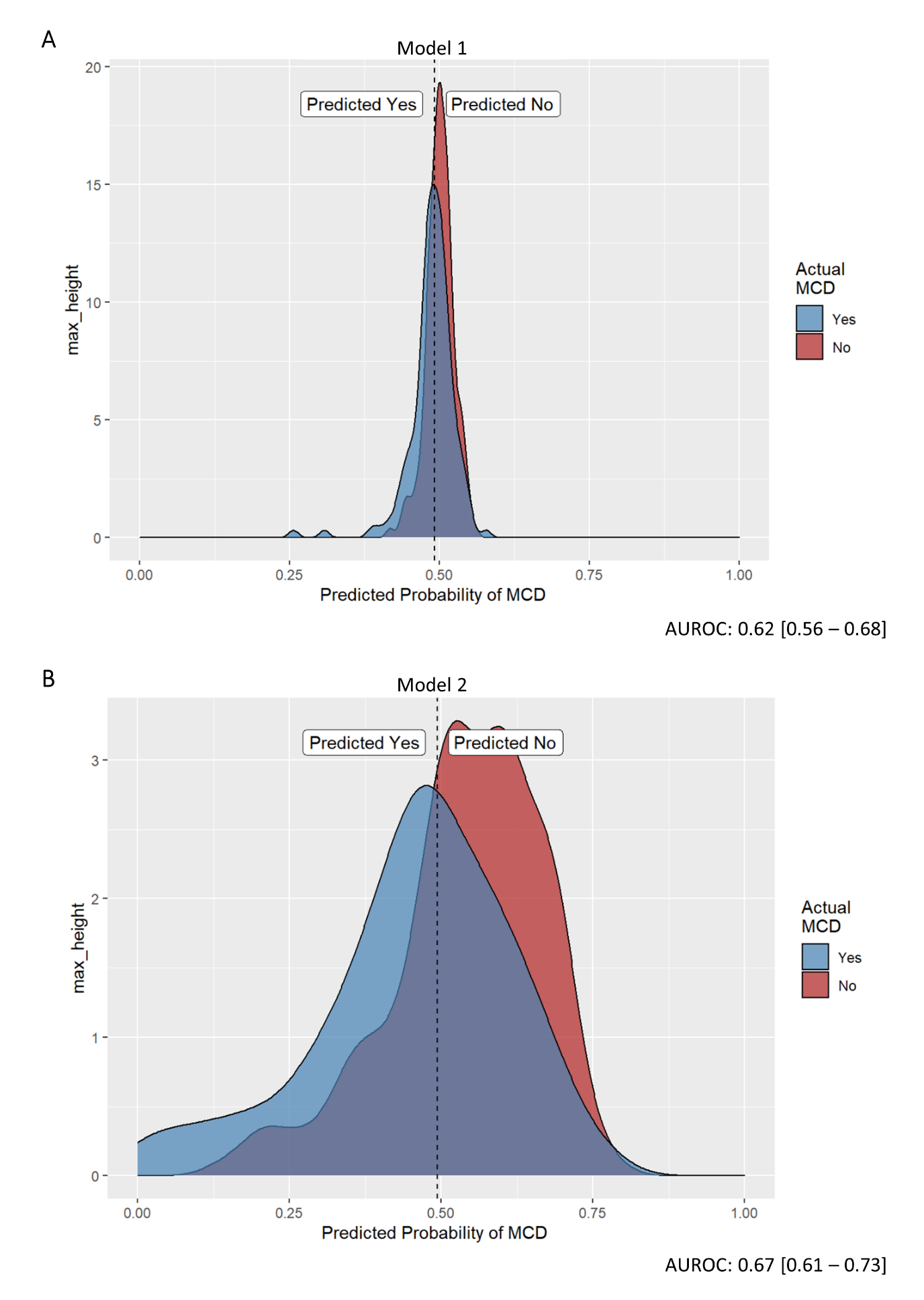


**Supplementary Fig. 1** Comparison of the distribution of the predicted probabilities showing the classification performance of the presence of MCD between two different models, being the x-axis the predicted probabilities for both classes and the y-axis the count of observations. **A**: Model 1 includes classical risk variables (age, sex, eGFR, NTproBNP, BMI, diabetes duration and systolic blood pressure >140mmHg). **B**: Model 2 includes classical risk variables and the NMR-assessed biomarkers. The inclusion of NMR parameters significantly increased the AUROC from 0.62 [0.56 – 0.68] to 0.67 [0.61 – 0.73], with a NRI considering NMR-assessed parameters of 21%. MCD: myocardial dysfunction, BMI: body mass index, NMR: nuclear magnetic resonance, AUROC: area under the ROC curve, NRI: net reclassification improvement.
